# Phosphoflow-Based Evaluation of Mek Inhibitors as Small-Molecule Therapeutics for B-Cell Precursor Acute Lymphoblastic Leukemia

**DOI:** 10.1371/journal.pone.0137917

**Published:** 2015-09-11

**Authors:** Aswathi A. George, Helicia Paz, Fei Fei, Jonathan Kirzner, Yong-mi Kim, Nora Heisterkamp, Hisham Abdel-Azim

**Affiliations:** 1 Division of Hematology/Oncology and Bone Marrow Transplantation, The Saban Research Institute of Children’s Hospital Los Angeles, Los Angeles, CA, United States of America; 2 Section of Molecular Carcinogenesis, Department of Pediatrics, The Saban Research Institute of Children’s Hospital Los Angeles, Los Angeles, CA, United States of America; 3 Leukemia and Lymphoma Program, Norris Comprehensive Cancer Center and Department of Pathology, University of Southern California, Los Angeles, CA, United States of America; University of Sydney, AUSTRALIA

## Abstract

Upstream mutations that lead to constitutive activation of Erk in B-cell precursor acute lymphoblastic leukemia (BCP-ALL) are relatively common. In the era of personalized medicine, flow cytometry could be used as a rapid method for selection of optimal therapies, which may include drugs that target the Erk pathway. Here, we evaluated the utility of phospho-flow, compared to Western blotting, to monitor Erk pathway activation and its inhibition by targeted Mek kinase inhibitors in human BCP ALL. Because the Erk pathway is not only activated endogenously, by mutations, but also by normal extracellular stimulation through stromal contact and serum growth factors, we compared Erk activation *ex vivo* in ALL cells in the presence and absence of stroma and serum. Phospho-flow was able to readily detect changes in the pool of pErk1/2 that had been generated by normal microenvironmental stimuli in patient-derived BCP-ALL cells passaged in NSG mice, in viably frozen primary patient samples, and in fresh patient samples. Treatment with the Mek1/2 inhibitor selumetinib resulted in a rapid, complete and persistent reduction of microenvironment-generated pErk1/2. Imaging flow cytometry confirmed reduction of nuclear pErk1/2 upon selumetinib treatment. An ALL relapsing with an activating KRasG12V mutation contained higher endogenous as well as serum/stromal-stimulated levels of pErk1/2 than the matched diagnosis sample which lacked the mutation, but selumetinib treatment reduced pErk1/2 to the same level in both samples. Selumetinib and trametinib as Mek inhibitors were mainly cytostatic, but combined treatment with the PI3K∂ inhibitor CAL101 increased cytotoxicity. Thus phospho-flow cytometry could be used as a platform for rapid, individualized *in vitro* drug sensitivity assessment for leukemia patients at the time of diagnosis.

## Introduction

Overall survival rates for childhood B-cell precursor acute lymphoblastic leukemia (BCP-ALL) using traditional chemotherapy have increased to more than 80%. However, prognosis at relapse is significantly worse, and a major effort involves identification of alternative therapies to treat such patients. Interestingly, Case et al [1] [2] reported that activation of the Ras pathway, which includes Raf, Mek and Erk, could be detected in 35% of diagnostic and 25% of relapsed samples. As reviewed in [3], because of “oncogene addiction”, cancers with constitutive activation of a specific signal transduction pathway are thought to be more sensitive to inhibitors of such pathway than cancers that lack constitutive activation.

Based on the finding of Ras pathway activation in many cancers and the lack of specific Ras inhibitors, there has been significant interest in the development of inhibitors that target components of this pathway downstream of Ras. These include small molecules that inhibit the kinase activity of Mek1/2 in the phosphorylation of Erk1 and Erk2, their only identified substrates [4]. Irving et al [5] recently applied this principle to test the non-ATP competitive Mek1/2 inhibitor selumetinib (AZD6244, ARRY-142886) as monotreatment for childhood ALL in preclinical studies and concluded that clinical evaluation of selumetinib is warranted.

The availability of a biomarker for selumetinib effectiveness would be very useful if this drug was to be tested on patients. Irving et al [5] cultured ALL cells without stroma for their *in vitro* studies on selumetinib and their discussion of Ras pathway activation centered on the intrinsic activation of Ras caused by genetic alterations. However, there are additional, extrinsic sources of Ras pathway activation that are not taken into account. The growth of primary BCP ALL, the persistence of minimal residual disease and relapse all take place under circumstances in which the cells are continuously exposed to and stimulated by serum-provided cytokines and growth factors. Moreover leukemia cells in the bone marrow associate with, and receive Ras pathway activating stimuli through multiple molecular interactions including contact with extracellular matrix (ECM) and stromal cells.

Thus the question of whether selumetinib is effective under such conditions of multiple sources of Ras pathway activation was not addressed. We standardly co-culture human ALL cells with protective stroma to model the circumstances found in the bone marrow microenvironment [6, 7]. In the current study, phospho-flow was used to investigate pErk levels as a surrogate marker for selumetinib effectiveness under such conditions of multiple sources of Ras pathway activation. We found that inhibition of extrinsic sources of Mek activation by selumetinib was rapid and persistent in some primary patient BCP ALL samples. Thus phospho-flow cytometry could be used as a platform for rapid, individualized *in vitro* drug sensitivity assessment for leukemia patients at the time of diagnosis using their fresh or frozen cells. This could contribute to decision-making in treatment strategies to add new, individualized targeted therapeutic agents to existing treatments. In turn, this may lead to improved outcome, allow de-escalation of standard chemotherapy and decrease the long-term side effects of therapy.

## Materials and Methods

### Cells and culture

The murine OP9 stromal cell line (CRL-2749) was obtained from the ATCC (Manassas, VA, USA). US7, US7R, TXL2 and ICN06 are patient-derived ALLs that have been passaged in NOD/SCIDγc-/- (NSG) mice (Jackson Labs, Bar Harbor, ME) and subsequently grown on OP9 stroma. US7, US7R, TXL2 and ICN06 have been previously described [8, 9]. US7 and US7R are also referred to as LAX7 and LAX7R [9, 10]. ICN06 was from a new diagnosis patient with a TEL-AML1 fusion protein. Primary patient LAX57 [t(1;9)(q44;p22)] (96% blasts: CD45+/-^(dim to negative)^ CD19+CD10+CD20+/-^(dim to negative)^ CD22+CD34+SIg-) was a new diagnosis sample whereas LAX56 [t(Y;7)(p11.3;p13)] (89% blasts: CD45+/-^(dim to negative)^ CD19+CD10+CD20+/-^(dim to negative)^ CD22+CD34+SIg-) was from a relapsed patient. These pre-B ALL cells grew directly out from Ficoll-purified bone marrow mononuclear cells when plated on irradiated OP9 stroma as described previously for primary BCP ALLs first passaged in NSG mice [11, 12]. Patient-derived ALLs were grown in αMEM medium supplemented with 20% FBS, 1% L-glutamine and 1% penicillin/streptomycin (Invitrogen Corporation). In all experiments, OP9 stroma was mitotically inactivated by irradiation. OP9 stromal cells did not contribute a signal to phospho-flow analysis of ALL cells: the irradiated stromal cell layers remain intact and adherent to the plate, while ALL cells are harvested by gentle pipetting. In addition, intact OP9 cells are very large, and are off-scale under the FSC/SSC voltage parameters used to gate on ALL cells (S1 Fig). Mononuclear cells from diagnosis patient samples were isolated using Ficoll and viably frozen (LAX56). Directly tested (non-cultured) samples include relapse LAX56 with 92% viability; a [t(1;4)(p22;q31)] which was 88% viable on day 2 (95% blasts: CD45+/-^(dim to negative)^ CD19+CD10+CD22+CD34+/-^(dim to negative)^ SIg-); LAX39 [t(12;21)(p13;q22)](TEL-AML1) (93% blasts: CD45+/-^(dim to negative)^ CD19+CD10+CD20-CD22+CD34+SIg-) with 78% overall viability; and LAX40 [t(8;14)(q24;q32)] (91% blasts: CD45+^(dim)^ CD19+CD10+CD20-CD22+CD34-SIg-) with 74% viability.

### Ethics statement

All human specimen collection protocols were reviewed and approved by Children’s Hospital Los Angeles Institution Review Board (IRB) [Committee on clinic investigations] (CCI).

Collections were in compliance with ethical practices and IRB approvals. All specimens were de-identified/anonymized before acquisition for research. ICN06 was collected after obtaining written informed consent; the rest of the specimens were collected as leftover specimens that were initially collected for clinical diagnostic purposes and were discarded as medical waste when no longer needed for clinical purposes. US7, US7R, TXL2 and ICN06 have been previously described [8, 9]).

### Phospho-flow analysis

Antibodies against Mek1/2 (pS218/pS22) conjugated to Alexa Fluor 647 (clone 024–836; #562460), and antibodies against Erk1/2 (pT202/pY204) conjugated to PE (clone 20A; #612566) were from BD-Biosciences. Clone 20A antibodies detected one pErk band on Western blots (not shown). Primary antibodies against Erk1/2 (pT202/pY204) (clone 197G2; #4377) from Cell Signaling Technology (CST), which detect two bands representing pErk1 and pErk2 on a Western blot as expected, were also used, in conjunction with secondary antibodies Biotin-SP-AffiniPure F(ab’)2 Donkey Anti-Rabbit IgG (#711-066-152; polyclonal) and Streptavidin-PE (#016-110-084) from Jackson Immunoresearch. For phospho-flow analysis with fluorochrome-conjugated antibodies, cells were immediately fixed after drug treatment with an equal volume of pre-warmed Cytofix (BD Biosciences #554655) for 10 minutes at 37°C. Cells were pelleted by centrifugation, resuspended by vortexing and permeabilized with chilled Perm Buffer III (BD Biosciences #558050). Cells were incubated on ice for 30 min, washed 3 times with Stain buffer (BD Biosciences #554656) and resuspended in a 50–100 μl volume for antibody staining in the dark, for 60 min at room temperature. For phospho-flow using unconjugated pErk1/2 in conjunction with secondary antibody and Streptavidin PE, cells were fixed, permeabilized and washed as above. Cells were then stained with a 1:200 final concentration of the primary antibody for 60 min in the dark, washed and stained with 1:200 dilution of the secondary antibody for 30 min, and finally with 1:100 dilution of Streptavidin PE. Phospho-flow cytometry data were acquired on a FACSCanto-II (BD Biosciences).

### Imaging cytometry analysis

Cells stained for phospho-flow as described above were used to study intracellular distribution of pErk using an Imagestream IS-100 (Amnis, EMD Millipore) flow cytometer. In addition to staining of pErk with PE, cell nuclei were stained using DRAQ5. Image files of 3500 to 20,000 events were acquired. To measure nuclear localization of pErk, we measured the similarity of the pErk and nuclear image pairs on a ‘per cell’ basis. The Similarity Score is a log-transformed Pearson’s correlation coefficient of the pixel values of the nuclear and pErk images. The greater the translocation of the pErk into the nucleus, the more similar the two images will be and result in a higher Similarity Score.

### Western blotting

Cells were lysed in SDS sample buffer (100 mM NaCl, 500 mM Tris, pH 8.0, 10% SDS) plus phosphatase inhibitors. Cell extracts were run on 4–20% NuPAGE Bis-Tris gels (Life Technologies, CA). Antibodies for Western blotting include: total Erk1/2 (Cell Signaling Technology #9102 polyclonal) and pErk1/2 (Thr202/Tyr204) (CST #4377 clone 197G2). We also tested pErk1/2 (Thr202/Tyr204) (clone 20A; BD #612358) but this only detected a single pErk band (not shown). pMek antibodies were from CST (#9154 clone 41G9; listed as against S217 and S221 both on Mek 1 and on Mek2). Gapdh (Genetex #100118; polyclonal) was used as a loading control. When indicated, membranes were stripped with Restore PLUS Western Blot Stripping Buffer (ThermoScientific #46430).

### Drug treatment

Details of stromal or FBS deprivation conditions are indicated in the figure legends and include culture without OP9 stroma but in αMEM medium supplemented with 20% FBS, 1% L-glutamine and 1% penicillin/streptomycin; culture without OP9 stroma in αMEM + 1% w/v BSA; and culture in chemically-defined serum-free X-Vivo15 medium (Lonza USA) + 1% BSA + L-glutamine. Trametinib, selumetinib and CAL101 were purchased from SelleckChem. Drugs were dissolved in DMSO and stored at -20°C. ALL cells were cultured in a 24-well plate at a density of 1x10^6^ cells/ml, in the presence or absence of irradiated OP9 cells. Cells were treated with various concentrations of trametinib, selumetinib or CAL101 for the time period indicated in each experiment. Cell viability and total cell number were measured by Trypan blue exclusion. A sodium orthovanadate 100 mM stock solution in dH_2_O with pH adjusted to 9.0 with HCL was boiled, cooled, re-adjusted to pH 9.0 and the procedure repeated. Aliquots were stored at -20°C. Cells were treated with 100 μM sodium vanadate for the indicated times.

### Statistical analysis

Statistical analysis was performed with Prism software. Data are presented as mean ± SD or mean ± SEM. Statistical significance of differences between groups was evaluated using one-way-ANOVA. Statistical significance of differences between two treatments was evaluated using Student’s t-test. The value of *p* < 0.05 was considered to be statistically significant.

## Results

**Fig 1:**
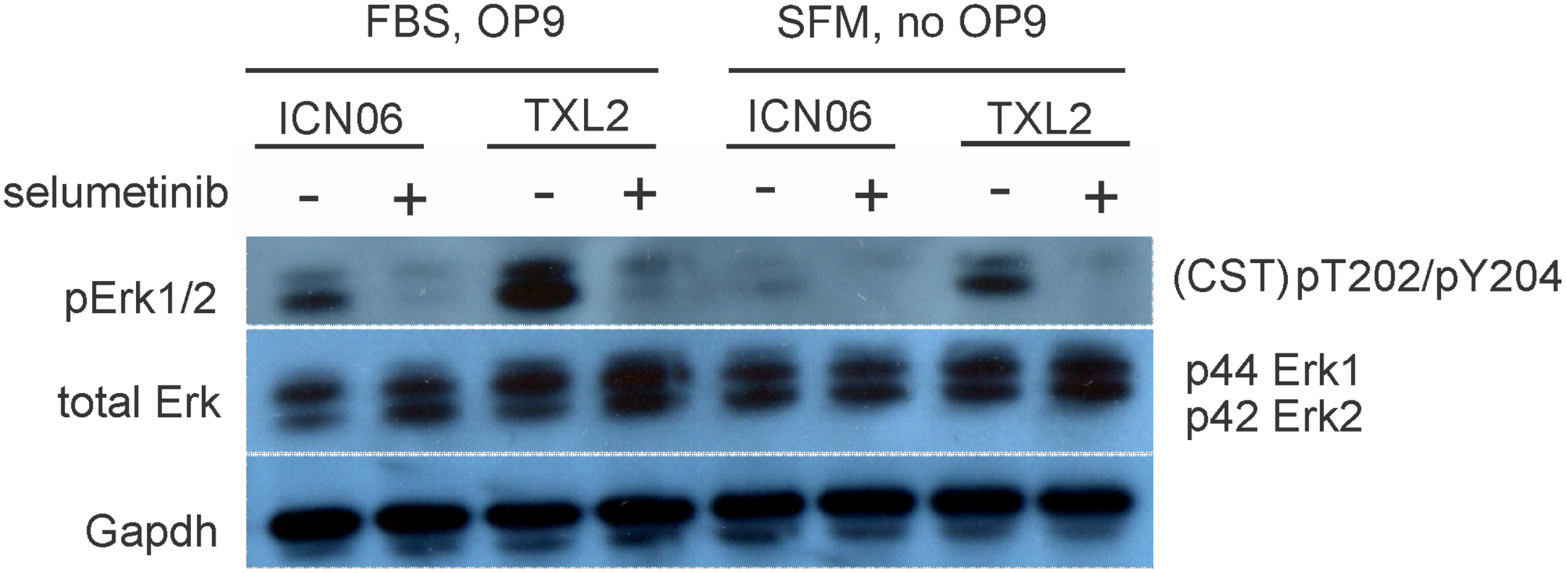
Western blot analysis shows pErk reduction through selumetinib treatment and by removal of stromal support. ICNO6 and TXL2 ALL cells were cultured for 4 hrs in αMEM + 20% FBS on OP9 stroma or in αMEM + 1% BSA without OP9 stroma, and then treated in the same media for an additional 4 hours with 10 μM selumetinib. Western blots were incubated with the antibodies indicated in the panel. Gapdh, loading control. The membrane was sequentially stripped and re-probed with antibodies.

We tested the effect of selumetinib on three different sources of Mek activation including the intrinsic, oncogene-driven pool, the Mek pathway activation through serum stimulation and Mek activation generated through contact with stromal cells. TXL2 (Ph-chromosome positive ALL) and ICN06 (TEL-AML1 fusion) were investigated as representative of two major subcategories of ALL. TXL2 contains the Bcr/Abl oncogene that, through its deregulated tyrosine kinase activity, constitutively activates the Ras pathway. Western blotting with CST pErk1/2 antibodies indicated that the ALL cells cultured with stroma contained higher levels of pErk1/2 than cells which did not receive stromal stimulation (Fig 1). A 4-hour treatment with selumetinib clearly reduced pErk1/2 originating both from serum as well as from stromal stimulation.

**Fig 2:**
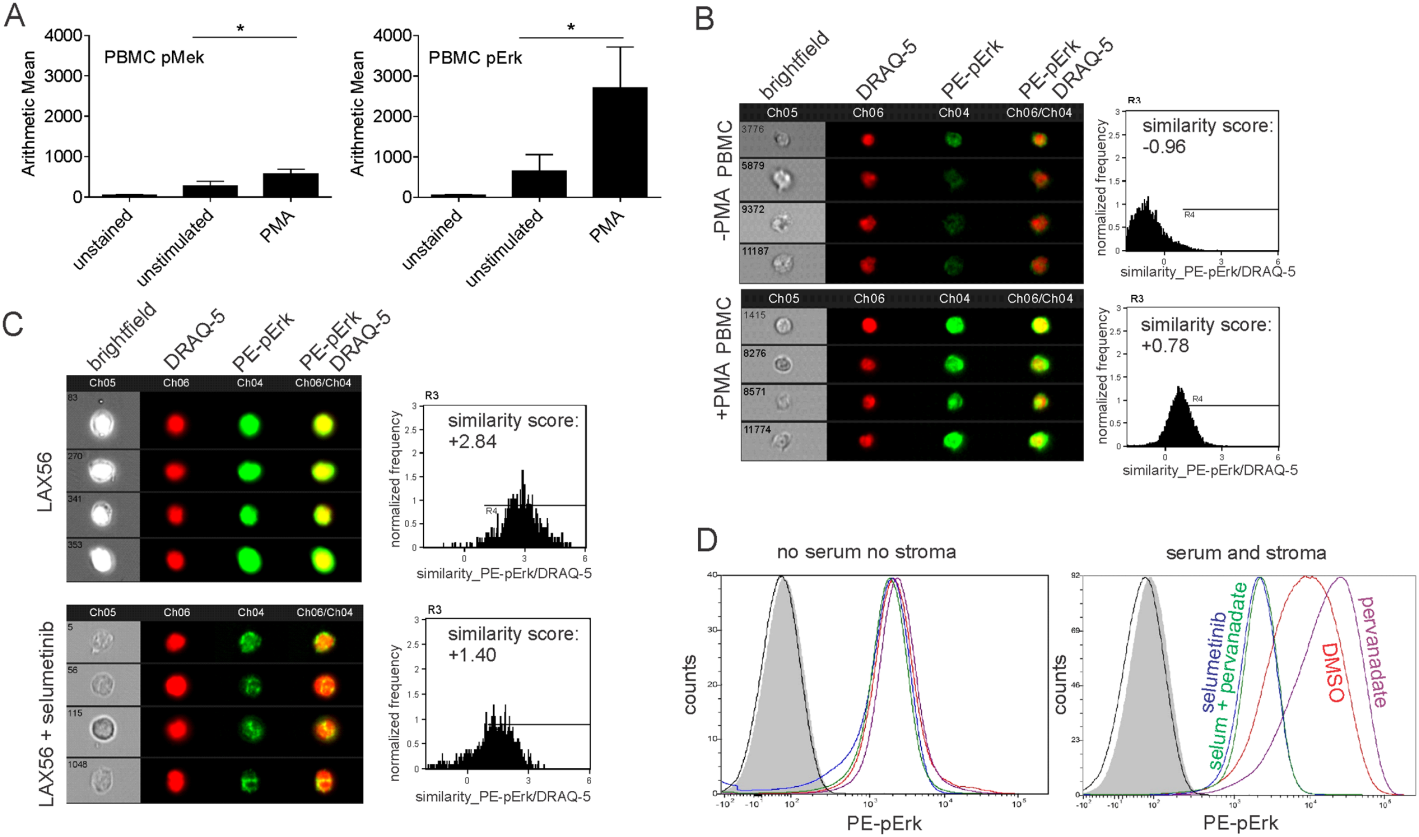
Phospho-flow cytometry detects Mek pathway activation and inhibition. (A) PBMC from normal donors were stimulated with 40 nM PMA for 15 minutes, then analyzed by phospho-flow. Left, pMek, right pErk. Error bars, mean ± SD of 3 independent samples. *p<0.05, Student's t-test. (B, C) Imaging cytometry analysis on PBMC not stimulated (B top) or stimulated with 40 nM PMA (B bottom) or LAX56 cells treated with DMSO (C, top) or 10 μM selumetinib (C, bottom) for 10 minutes. (B, C) left, representative images; right, summary plot. Analysis of 3500 to 20,000 events per condition; numbers in the brightfield images represent the number designation of the individual event shown. (D) Histograms of pErk1/2 phospho-flow analysis on TXL2 ALL cells cultured without stroma or FBS (left panel), or on OP9 stroma + 20% FBS (right panel), for 24 hours. Cells were treated for 4 hours with solvent DMSO (red), with 100 μM sodium pervanadate (purple), with 10 μM selumetinib (blue), or with 100 μM sodium pervanadate, plus 10 μM selumetinib (green) as indicated. Grey histograms, unstained controls; black line, secondary antibody only. Single analysis.

FACS analysis, which would allow drug evaluation on small clinical samples, would be useful for monitoring the effects of Mek inhibitors such as selumetinib in patients. We first determined the sensitivity of flow cytometry to detect changes in pErk and pMek. Panel A in Fig 2 shows that phospho-flow was able to detect increases in pMek and pErk1/2 in normal PBMC stimulated by exposure to PMA, a very strong extrinsic activator of the Ras pathway. Since phosphorylated Erk1/2 functions as a transcription factor and migrates to the nucleus, we determined if this could be tracked using imaging flow cytometry. PBMC were stimulated or not with PMA, and compared by Imagestream analysis. Panel B in Fig 2 shows that nuclear translocation of pErk can be visualized, and the result quantified as similarity score, using this technology.

We next tested if pErk relocation was also detectable in a steady-state ALL, LAX56, growing in serum and on stroma, after the cells had been treated with DMSO or 10 μM selumetinib for 10 minutes. Interestingly, non-drug treated LAX56 cells contained nuclear pErk1/2 and when we treated the cells with selumetinib, levels of nuclear pErk1/2 were clearly reduced (panel C in Fig 2).

We also determined the magnitude of pErk1/2 detection possible with phospho-flow in such samples. We used TXL2 cells which maintain full viability for 24 hours when kept without serum or stromal cells (not shown). As shown in panel D in Fig 2, the removal of all sources of external Mek pathway activation resulted in cells with levels of pErk1/2 that could not be further reduced by treatment with selumetinib. Consistent with results in Fig 1 and panel C in Fig 2 phospho-flow measured very high levels of pErk1/2 in the ALL cells when they were grown with serum and stroma. To determine if this is the maximal achievable level, we also treated the cells with sodium pervanadate, a phosphatase inhibitor that causes accumulation of pErk1/2. Pervanadate slightly increased pErk levels beyond that which was already present in TXL2 ALL cells grown with stroma and full serum (panel D in Fig 2) but selumetinib was able to reduce all pErk1/2 to baseline pErk as observed in TXL2 cells cultured in the absence of stroma or serum. Together, these results validate phospho-flow as a readout for selumetinib inhibition of Mek pathway activation.

**Fig 3:**
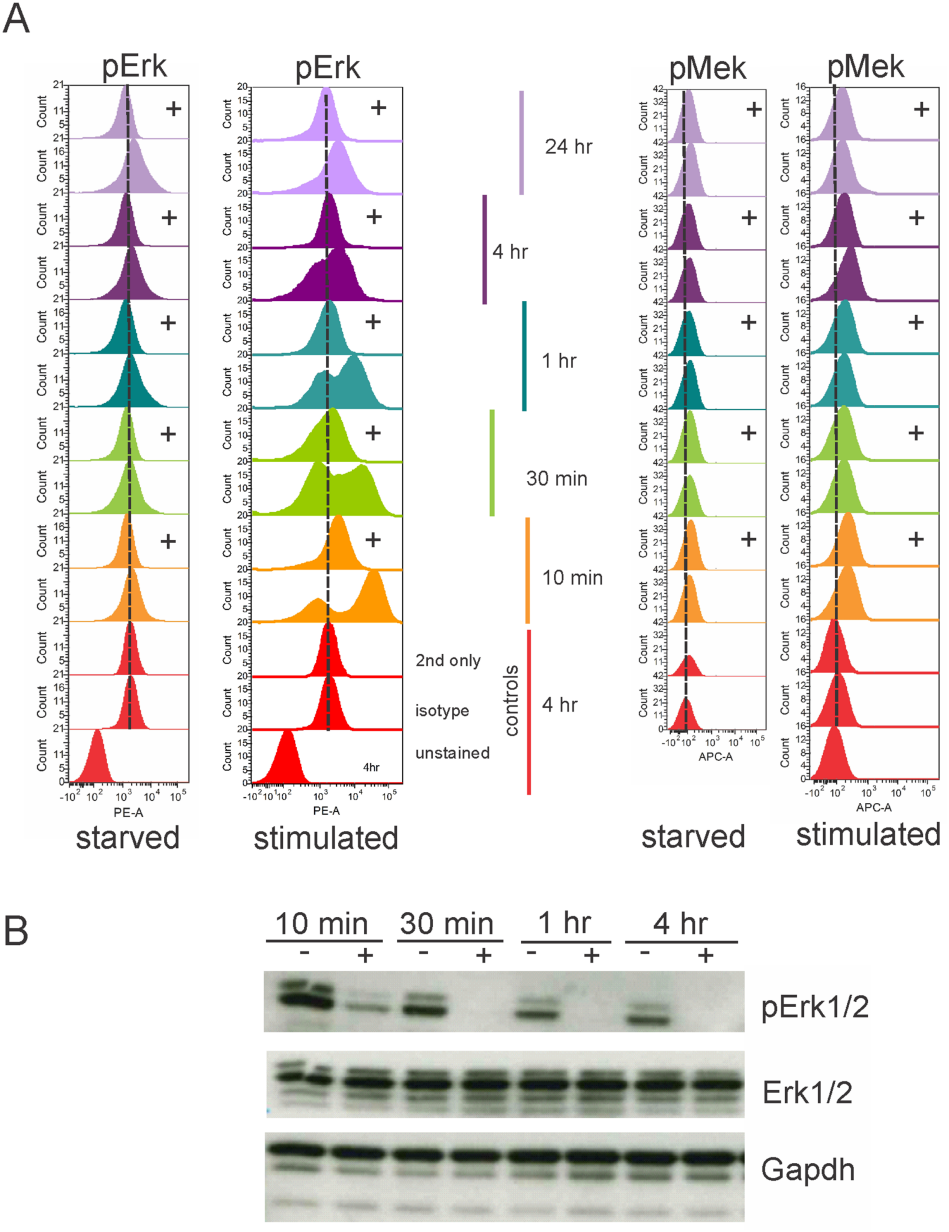
Phospho-flow measures long-term inhibition by selumetinib of pErk generated in BCP-ALL cells by extracellular serum stimulation. TXL2 ALL cells, made maximally quiescent by culture in αMEM + 1% BSA for 24 hours without OP9 stromal support or FBS (marked ‘starved’ below panel), were stimulated by 20% serum addition and stroma at t = 0 (marked 'stimulated' below panel), while exposed (+) or not (no symbol) to 10 μM selumetinib. Phospho-flow using pErk1/2 (CST) in (A) left part of panel or pMek (BD) antibodies in (A) right part of panel taken at the indicated time points. (B) Western blot analysis for pErk1/2. One of two experiments with similar results. Gapdh, loading control. The membrane was sequentially stripped and reprobed with antibody.

To further test the ability of selumetinib to suppress Mek-induced pErk1/2, we generated deeply quiescent TXL2 cells by overnight deprivation of stromal support and serum, then added the ALL cells to OP9 stromal cells in complete (containing serum) medium in the presence or absence of selumetinib. As shown in panel A in Fig 3, activation of the Mek pathway by external stimulation was rapidly detectable, within 10 minutes, as a marked increase of pErk1/2 by phospho-flow. This was confirmed by Western blotting (panel B in Fig 3). Selumetinib rapidly reduced the *de novo* generated pErk1/2, and inhibition of Mek activity persisted at least for 4 hours (panel A in Fig 3). Western blotting for pErk1/2 confirmed this result (panel B in Fig 3). In comparison to stimulated cells, the pool of pErk1/2 left after starvation as measured by phospho-flow showed only a small reduction in the samples treated with selumetinib (panel A in Fig 3). This result shows that endogenous levels of pErk are low, with most of the activated Erk generated by external stimuli.

We also investigated pMek in the same cells. In concordance with the low endogenous Erk1/2 activation in starved ALL cells, levels of upstream activated Mek were also very low. When the cells were stimulated with serum, pMek levels increased to a small degree. Since selumetinib inhibits the kinase activity of Mek and not the upstream phosphorylation of Mek, no effect of selumetinib on pMek levels was expected or found (panel A in Fig 3).

**Fig 4:**
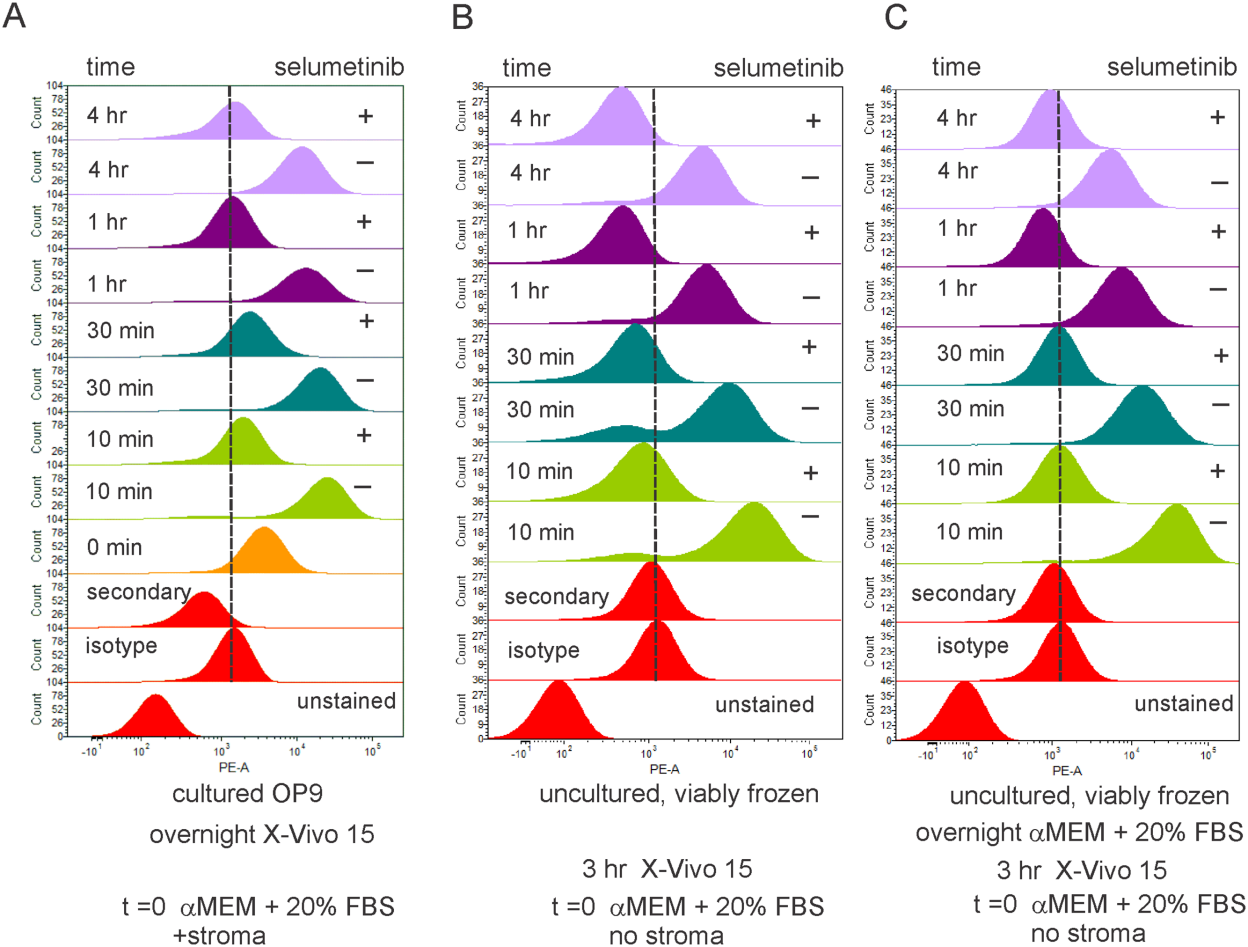
Selumetinib inhibitability of pErk1/2 generation is a stable characteristic of ALL cells. Primary LAX56 cells expanded long-term on OP9 stroma (A) or non-cultured viably frozen and thawed samples (B, C) were examined for selumetinib-inhibitable pErk1/2 generation using pErk1/2 (CST) antibodies. Pre-treatment details are indicated below the panels. At t = 0, cells were stimulated with 20% FBS in αMEM and stroma for the indicated time in the absence (-) or presence (+) of 10 μM selumetinib. Single sample, three different conditions (A-C).

TXL2 ALL cells, although not established as a traditional leukemia suspension-growing cell line, were passaged in NSG mice before being cultured with stromal support OP9 cells. We therefore considered the possibility that the marked reaction of TXL2 cells to extrinsic Erk pathway stimulation, and its inhibition by selumetinib, were a consequence of extensive *ex vivo* manipulation. To address this, aliquots of LAX56 cells, which were not passaged in NSG mice and grew directly on OP9 stroma, were compared to original LAX56 cells viably frozen at diagnosis. Fig 4 demonstrates that all samples, irrespective of treatment history, generated a surge of pErk within 10 minutes of serum stimulation, which was inhibitable for at least 4 hours by one administration of 10 μM selumetinib. The results also indicate that selumetinib-inhibitable Mek pathway activation by serum stimulation is an inherent characteristic of this BCP ALL patient sample.

**Fig 5:**
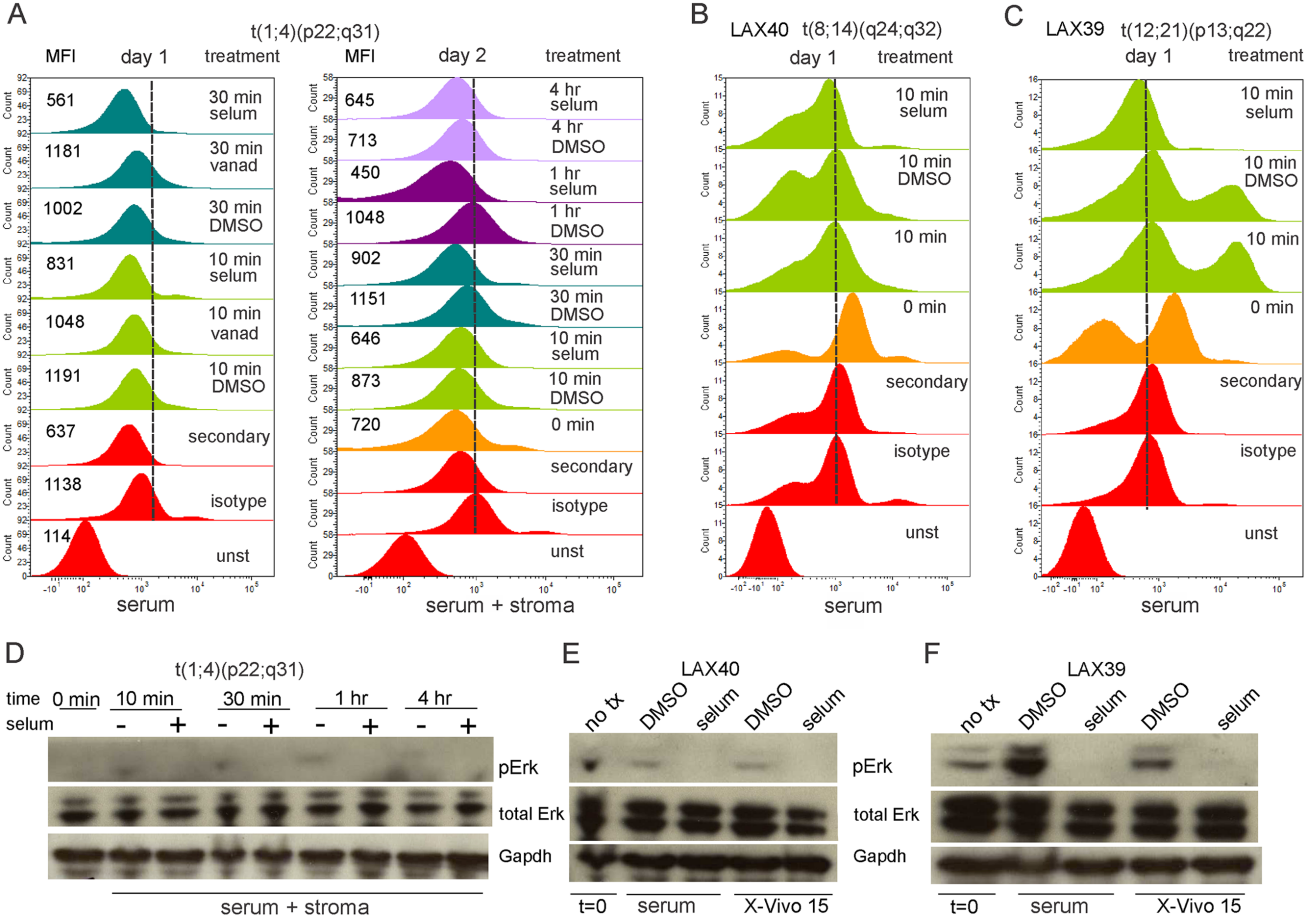
Diagnosis ALL samples differ in ability to activate Erk1/2 upon serum stimulation. Diagnosis sample t[(1;4)(p22;q31)](A, D); LAX40 [t(8;14)(q24;q32)] (B, E); or LAX39 (TEL-AML1) (C, F) were assayed for serum-stimulated generation of pErk1/2 (CST antibodies) by phospho-flow (A-C) or Western blot (D-F) in the presence or absence of selumetinib. (A) Cells were directly stimulated with 20% FBS in αMEM (day 1, left panel) or placed overnight in X-Vivo15 prior to stimulation with 20% FBS and stroma (day 2, right panel) for the indicated period of time in the presence of solvent DMSO or 10 μM selumetinib. (B, C) ALL cells in X-Vivo15 medium for 3 hrs were stimulated with 20% FBS in αMEM in the presence of 10 μM selumetinib or DMSO as indicated. MFI, mean fluorescent intensity. (D, E, F) Samples harvested from the same cultures used for phospho-flow. (D) Western blot on day 2 samples. (E, F) serum αMEM; 20% FBS in αMEM. Three different diagnosis samples, each analyzed once.

We next analyzed three other original diagnostic BCP ALL samples. Interestingly, BM ALL cells directly after Ficoll isolation generated a minimal amount of pErk when stimulated with serum, and pervanadate treatment did not result in accumulation of pErk (panel A in Fig 5). Overnight incubation in X-Vivo15 medium followed by serum stimulation yielded a similar result (panel A in Fig 5). A second sample, LAX40, containing a translocation typical for Burkitt lymphoma, similarly did not respond to serum stimulation (panel B in Fig 5). Importantly, LAX40 also contained 4% normal residual lymphocytes that responded to the serum stimulation with increased pErk1/2 generation which was inhibitable with selumetinb (panel A in S2 Fig). This indicates that the leukemia cells in the sample were selectively unresponsive to serum stimulation. LAX39, a TEL-AML1-positive ALL sample contained two populations of leukemia cells (panels B and C in S2 Fig), one that responded vigorously to a 10-minute serum stimulation and one that reacted minimally (panel C in Fig 5).

Parallel Western blot analysis confirmed the overall lack of responsiveness of the total cell populations (panels D, E in Fig 5). The pErk1/2 present and induced in sample #39 that was detected by phospho-flow, and its inhibitability with selumetinib, was confirmed by Western blotting (panel F in Fig 5). These results show that selumetinib rapidly and effectively reduces downstream Erk1/2 phosphorylation if it can be generated through external stimuli.

**Fig 6:**
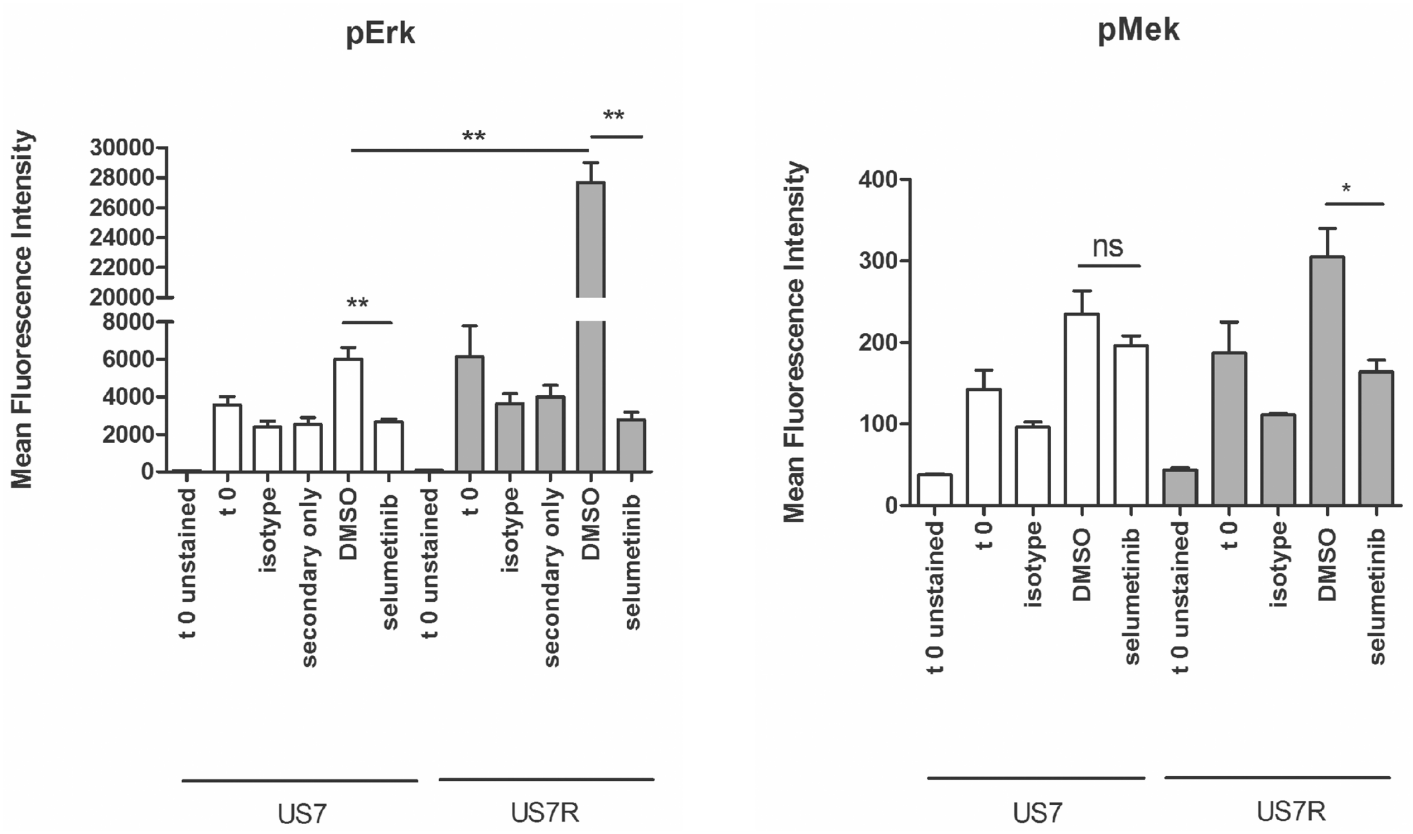
Phospho-flow detects increased pErk1/2 in US7R relapse sample with an activating K-Ras mutation compared to matched diagnostic US7 sample lacking the mutation. Cells starved overnight in X-Vivo15 medium were stimulated for 4 hours with αMEM + 20% FBS and OP9 stroma in the presence of solvent DMSO or 10 μM selumetinib. Controls (t 0 samples) continued to be starved in αMEM with 1% BSA in the absence of stroma. US7 and US7R cells were assayed for serum-stimulated generation of pErk1/2 by phospho-flow using pErk1/2 (CST, left panel) or pMek (BD, right panel) antibodies. Error bars, mean +/- SEM of three independent experiments. *p<0.05 **p<0.01. Student's t-test.

We also were able to compare one matched diagnosis and relapse sample from the same patient (Fig 6). The diagnosis sample US7 is wild type for K-Ras, whereas the relapse had acquired an activating KRasG12V mutation [10]. Phospho-flow showed a difference that was not statistically significant in endogenous (t = 0 starved samples) pErk1/2, that became almost 4-fold higher in the US7R K-RasG12V sample upon stimulation with serum and stroma (DMSO samples). Interestingly, whereas the concurrent treatment with selumetinib was able to suppress pErk1/2 production by half in the diagnostic sample, it was also surprisingly effective in reducing the elevated pErk1/2 in the relapse sample to background levels.

**Fig 7:**
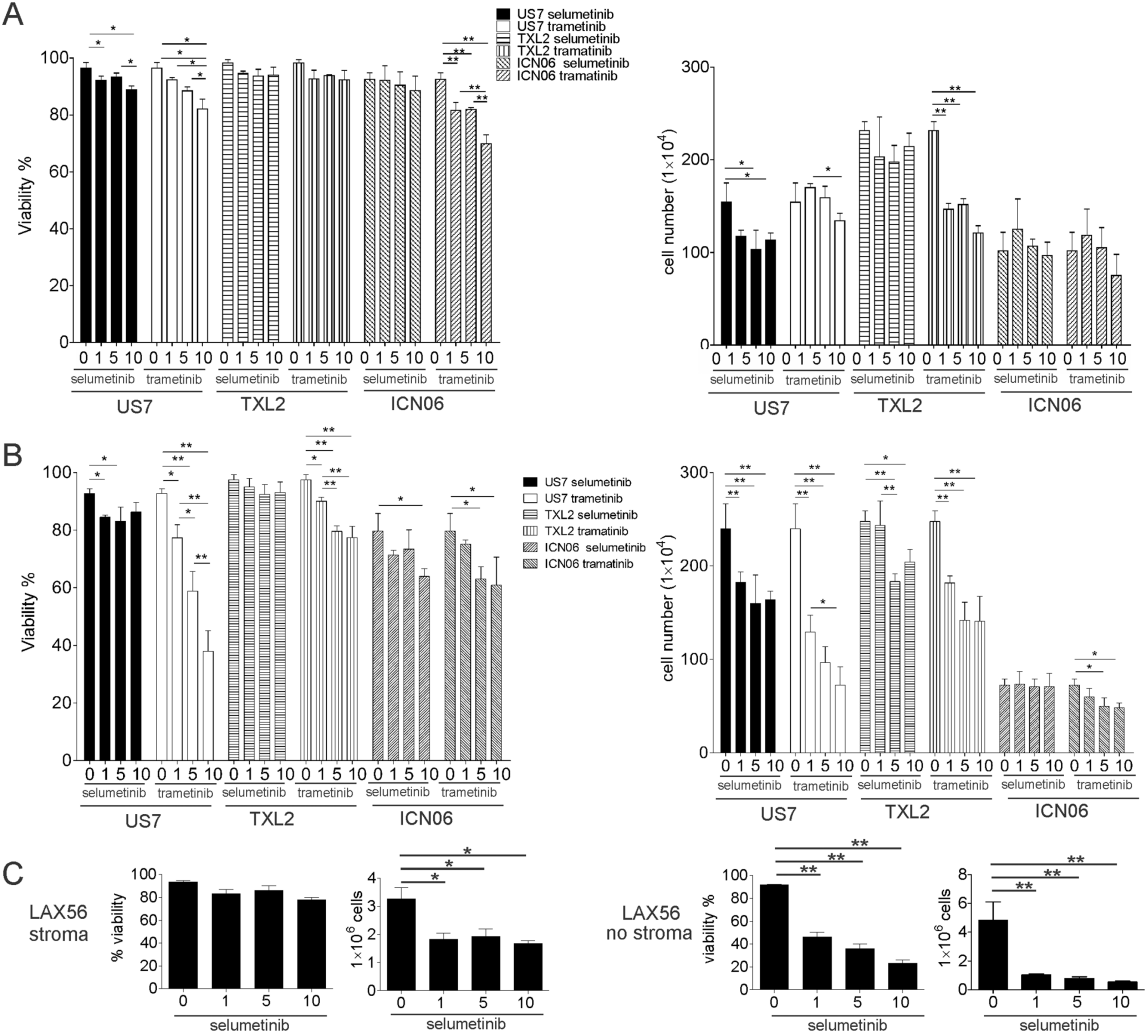
Evaluation of selumetinib and trametinib Mek1/2 inhibitors as mono-treatment on BCP-ALL cell viability in the presence and absence of stroma. The indicated ALLs were treated with different μM concentrations of selumetinib or trametinib for 72 hours in the presence (A) or absence (B) of irradiated OP9 stromal support. Note: “0” values were determined on one sample set but are shown twice, for selumetinib and trametinib, for clarity. (C) 72-hour treatment of primary LAX56 with selumetinib as indicated. Viability (left panels) and cell counts (right panels) were determined by Trypan Blue exclusion. Error bars, SD of values measured in triplicate wells. One of two experiments for US7 and TXL2 with similar results. *p<0.05 **p<0.01, one-way ANOVA.

We next measured the outcome of Mek inhibition on cell viability and proliferation using TXL2 and ICN06 cells treated with selumetinib in the presence of stroma and serum. We also included trametinib (GSK1120212, JTP-74057), a chemically distinct allosteric Mek1/2 inhibitor, and US7 in this analysis. Even a 3-day exposure to the highest dose of 10 μM selumetinib did not have a pronounced cytotoxic effect on these ALLs. Cell proliferation was slightly reduced, compared to non-drug-treated controls (panel A in Fig 7).

To examine the contribution of OP9 stromal-induced Erk1/2 activation on Mek1/2 inhibitor activity, we removed the stromal support when treating the ALL cells with inhibitors. However, even under these circumstances, viability of ICN06 and TXL2 was largely maintained, although US7 cells became more sensitive to the cytotoxic and cytostatic effects of the Mek1/2 inhibitors (panel B in Fig 7). We also tested combinations of selumetinib and trametinib but found no added benefit of this on cell viability or proliferation in the presence or absence of stroma (results not shown). Selumetinib was cytostatic for primary LAX56 ALL cells and cytotoxic when these cells were not supported by stroma (panel C in Fig 7).

The only known substrates of Mek1/2 are Erk1/2, but Erk1/2 may be activated through other upstream pathways. Inhibition of PI3Kδ with CAL101 (idelalisib) was shown to strongly inhibit phosphorylation of Erk1/2 in multiple myeloma cells [13]. To attempt to further reduce levels of endogenously activated Erk, we treated US7 and TXL2 cells with trametinib, CAL101 or a combination of both, in the presence and absence of stroma and measured the effect on pErk levels.

**Fig 8:**
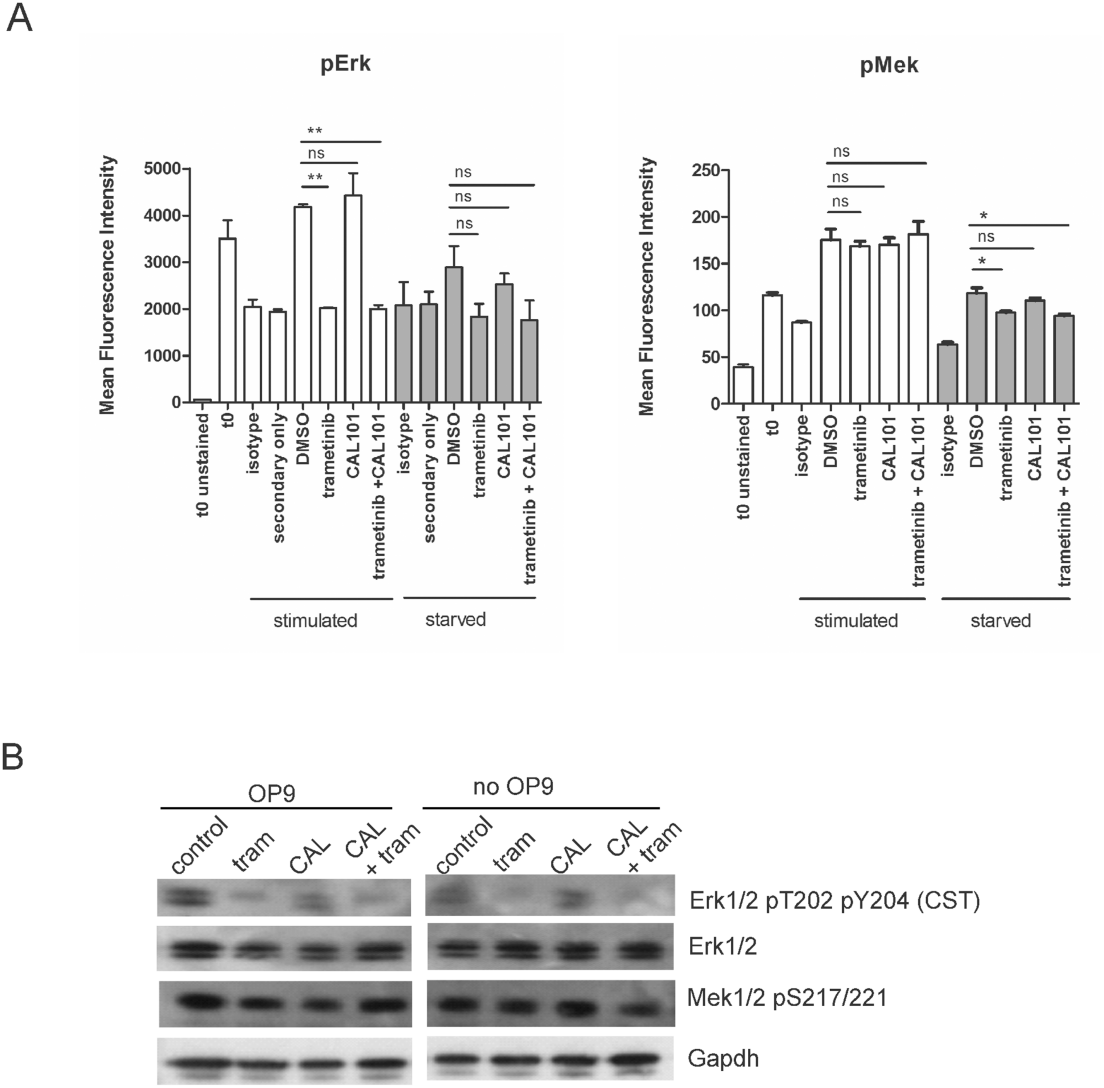
Mek pathway inhibition in combination-treated BCP-ALL cells by phospho-flow and Western blot. (A) Phospho-flow on US7 cells using pErk1/2 (CST) or pMek (BD) antibodies in the left and right panels respectively, as indicated, in the presence or absence of trametinib, CAL101 or both. All samples were starved overnight in X-Vivo15 medium (t 0 samples), then treated from t 0 onward for 4 hours with αMEM + 20% FBS and OP9 stroma (‘stimulated’), or with αMEM + 1% BSA in the absence of stroma (‘starved’). At t 0 solvent DMSO or 10 μM of the indicated drugs was also added to the samples. Error bars, mean +/- SEM of three independent experiments. *p<0.05 **p<0.01, Student's t-test. (B) Western blot analysis (single analysis) of US7 cells grown for 24 hours in αMEM + 20% FBS and OP9 stroma (‘OP9’ samples, left panel), or in αMEM + 1% BSA in the absence of stroma (‘no OP9’ samples, right panel). Cells were then additionally treated for 4 hours with solvent DMSO (control) or 10 μM of the indicated drugs. Gapdh, loading control. Western blot membrane was sequentially stripped and reprobed with antibodies.

Panel A in Fig 8 illustrates that while treatment with trametinib significantly reduced pErk1/2 to background levels, CAL101 treatment failed to reduce pErk1/2. Also, combination treatment did not further decrease the level beyond that seen by trametinib treatment alone. Starved cells showed the same pattern of change in pErk1/2 levels, albeit with more modest effects. Western blotting confirmed reductions in pErk1/2 in drug-treated ALL cells (panel B in Fig 8). pMek levels as determined by phospho-flow showed little change as a result of drug treatment, beyond the increase over quiescent levels seen as a result of serum and OP9 stimulation, which was as expected.

**Fig 9:**
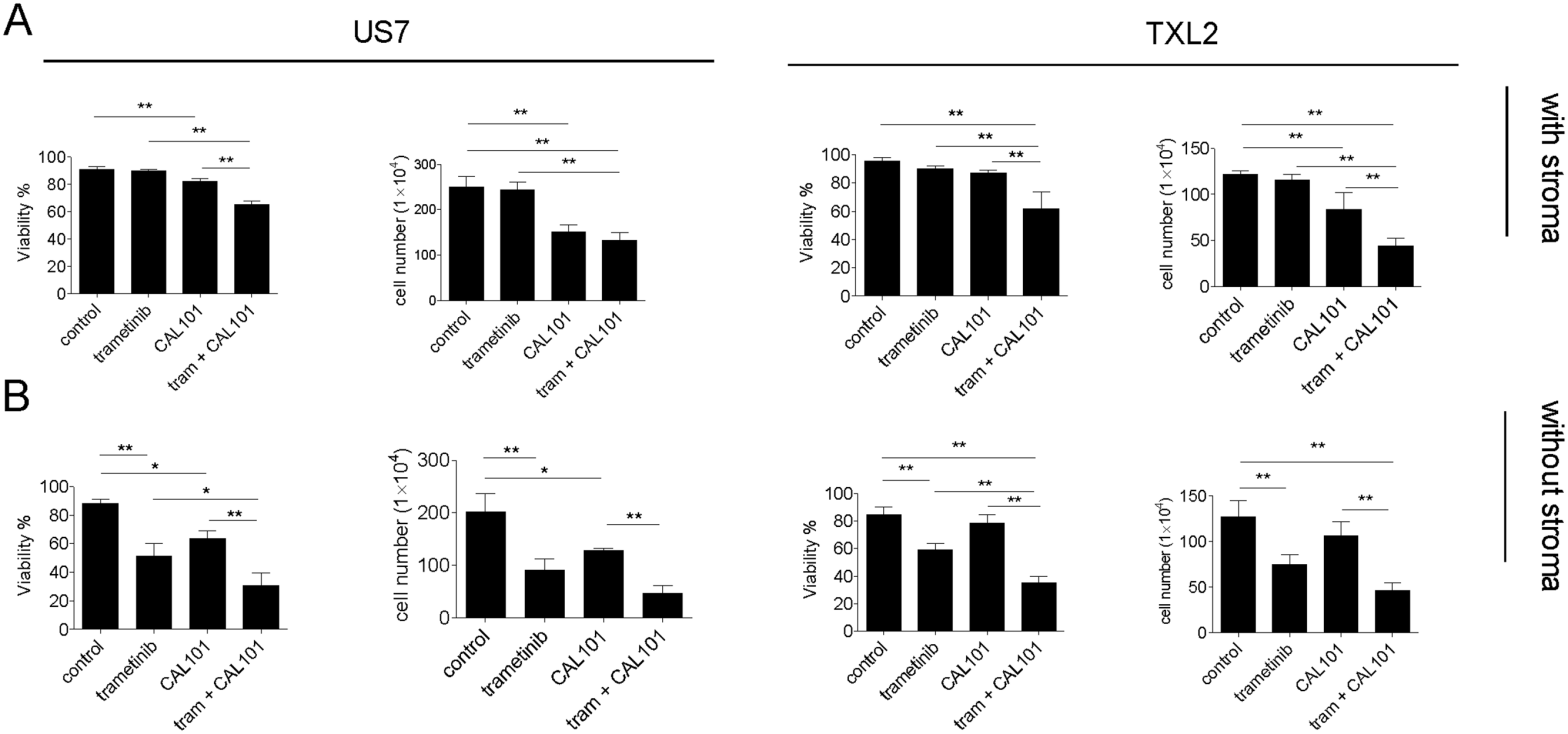
Viability and proliferation of ALL cells treated with trametinib and CAL101. US7 or TXL2 cells (10^6^ cells) as indicated were treated for 72 hours with 10 μM trametinib, 10 μM CAL101, or a combination of the two. Viability and cell counts were determined by Trypan blue exclusion. Cells were either exposed to drug while on stroma (A) or without stroma (B) in complete medium with 20% FBS. Error bars, mean ±SEM of triplicate values. *p<0.05; **p<0.01, one-way ANOVA.

A 3-day exposure to 10 μM of either drug as monotreatment in the presence of stroma had only small effects on viability, although CAL101 was cytostatic. The combination of both drugs was cytotoxic and cytostatic even when stromal support was provided (panel A in Fig 9), and this was enhanced when cells were treated with these drugs in the absence of stroma (panel B in Fig 9). We conclude that unlike in multiple myeloma cells, inhibition of PI3Kδ with CAL101 in ALL cells does not strongly inhibit generation of pErk1/2. Nonetheless CAL101 activity on ALL cells alone or combined with Mek inhibitors warrants further investigation.

## Discussion

Although many drugs in clinical development are inhibitors of protein kinases, few published studies to date report using phospho-flow for analysis of inhibition of constitutive or induced levels of intracellular signal transduction proteins in patient samples. In multiple myeloma and pediatric acute myelogenous leukemia, levels of constitutive and cytokine-induced pStat3 and pStat5 were evaluated as prognostic factors for outcome. Interestingly, those studies reported that a response to IL6 stimulation correlated with a better outcome [14, 15]. Perl *et al* [16] reported that sirolimus treatment in AML patient samples could be monitored using flow cytometry and antibodies against phosphorylated S6 ribosomal protein.

In addition to the study of Irving *et al* [5], the technology of phospho-flow has been applied earlier by Gregorj *et al* [17] to show that high levels of pErk1/2 in diagnosis ALL patient samples correlate with a high WBC, but that Ph-positive ALL do not contain significantly higher levels than the other samples. Analysis of Mek induction and inhibition in BCP-ALL samples using phospho-flow had not been reported and our study is thus the first to show that changes in pErk1/2 can be reproducibly detected using this method, if the ALL cells initially contained biologically relevant (i.e., other than through artificial PMA stimulation) levels of pErk1/2. Phospho-flow showed, and Western blotting confirmed, that pErk1/2 is clearly detectable *ab initio* in ALLs when these are cultured with serum and provided with stromal support. This was not only measured in Ph-positive TXL2 ALL cells but also in ICN06 and US7 as well as in two BCP ALL samples, LAX57 and LAX56, primary patient samples that grew directly on OP9 stroma (S3 Fig). Such circumstances can be regarded as modeling the bone marrow microenvironment, in which the Mek/Erk pathway in leukemia cells is continuously stimulated by the presence of serum and cell-cell contact. Even under these conditions, exposure to selumetinib clearly inhibited Mek activity as measured by a reduction of dually phosphorylated Erk1 and Erk2. We also found that phospho-flow could be used as a sensitive and rapid assay to monitor pErk1/2 generated by stimulation of quiescent cells with serum, and showed that the *de novo* generation of Erk1/2 was inhibitable by selumetinib.

A comparison of a paired set of diagnostic and relapse samples from the same patient (US7 and US7R respectively) using phospho-flow yielded some interesting observations. US7 is wild type for KRas, and based on Irving *et al* [5] would not be expected to show much sensitivity to selumetinib. Phospho-flow nonetheless detected pErk levels above baseline that were reduced by selumetinib treatment. The US7R cells show a significant 4-fold increase in serum/stromal stimulated pErk1/2 expression, most likely a result of the activating KRasG12V mutation acquired at relapse. Thus if analysis on a larger set of non-Ras mutated ALL samples using the methodology applied here reveals that ‘normal’ serum starved and stimulated pErk1/2 values exist in ALL samples, finding high pErk levels at diagnosis or relapse in specific samples could be an indication for sequencing the samples for mutations in components of the Ras pathway.

Interestingly, the leukemic cells in two primary BCP ALL samples, one of which (LAX40) was characterized by a Myc translocation more commonly seen in Burkitt lymphoma, did not contain significant constitutive levels of pErk1/2, nor was pErk1/2 inducible when the cells were stimulated with serum. In concordance with this, the leukemia cells also did not react when exposed to selumetinib. Also, phospho-flow for pErk1/2 showed that a third BCP ALL sample contained two populations of cells of which only the larger cells, which constituted around 35% of the population (panels B, C in S2 Fig), generated pErk1/2. These results show that heterogeneity exists among ALLs with respect to Mek activation, and further studies will be required to determine its causes and possible consequences.

As emphasized by Chappell *et al* [18], inhibitors of the Erk pathway can be classified as cytostatic but not as cytotoxic anticancer drugs: by selectively inhibiting the activity of their targets, such drugs suppress the proliferation of tumor cells that have constitutive activation of a pathway but, as monotreatment, do not kill target cells. In concordance with this, although we could clearly measure a biochemical effect of Mek pathway inhibition, the biological effect on three different BCP ALLs was much less pronounced. Thus we concur with Chappell *et al* [18] that the activity of Mek inhibitors is at best cytostatic. Also, we were not able to detect a clearly increased, or unique sensitivity of TXL2 towards either selumetinib or trametinib compared to US7 and ICN06, as would be predicted based on the ‘oncogene addiction’ habit of TXL2.

Irving *et al* [5] recently reported testing selumetinib in mice transplanted with human ALL cells. Although they noted decreased numbers of leukemia cells in the peripheral blood and spleen of the drug-treated mice, survival was not reported nor data describing bone marrow involvement or leukemia cell percentages in treated and non-treated mice. We found a moderate cytostatic effect and cytotoxicity for US7 when BCP ALL cells were without stroma but in the presence of serum, as a model for ALL cells in the circulation. However ALL cells protected by stroma were not sensitive to trametinib and based on these results we would predict that this inhibitor would have little effect on minimal residual disease in the bone marrow of patients.

Neither Western blotting for phospho-proteins nor phospho-flow is quantitative, and we therefore cannot determine unambiguously if trametinib or selumetinib-treated cells contain residual, low levels of phosphorylated Erk1/2, which could maintain their viability. Also, it is unknown whether or not leukemia cells can be killed even if phosphorylation of Erk1/2 could be completely eliminated. To further reduce Erk pathway-mediated survival signals, we tested a combination of trametinib with CAL101 (idelalisib) as two available, FDA-approved inhibitors (for melanoma and relapsed chronic lymphocytic leukemia, respectively). With the highest concentrations of both drugs tested (10 μM) we were able to reduce cell numbers of US7 and TXL2 to about 50% of control after 3 days of treatment. Interestingly, Wang *et al* [19] recently reported testing a new selective PI3Kδ inhibitor for treatment of BCP ALL. This compound reduced Erk1/2 signal transduction by inhibition of an atypical PI3Kδ-PDK1-Mek1/2Erk1/2 pathway. Although this drug was not tested on ALL cells grown with stroma, it suggests that treatment of BCP ALL with other inhibitors that efficiently target more than one upstream source of Erk1/2 activation may be more useful than the use of inhibitors that only target Mek1/2. An alternative, entirely novel approach discussed by Shojaee *et al* is to sequentially treat BCP ALLs with Mek pathway inhibitors and inhibitors of Mek pathway phosphatases [9, 10].

In the era of personalized medicine, flow cytometry could be a useful platform to assess the effects of new drugs on inhibition of pathways that affect leukemic cell survival and proliferation. Phospho-flow has obvious advantages over traditional Western blotting including speed, the need for only limited amounts of cells, the ability of studying heterogeneous sub-populations defined by immunophenotypic markers and the ability for single cell measurements of phosphorylated proteins. Our results clearly illustrate all these advantages and indicate that a relatively rapid assay (same-day results) can be used to measure reduction in pErk1/2 in ALL cells after treatment with targeted kinase inhibitors such as selumetinib. Recently, a differential sensitivity between BCP ALLs with and without a functional pre-B cell receptor to Mek and Erk1/2 inhibitors was reported [9, 10]. This further supports the concept that methods such as those used in the current study, which empirically determine drug-induced inhibition of constitutive and inducible pErk levels, may be useful in the future to guide clinical treatment.

## Supporting Information

S1 Fig Overlay of signals generated by harvested irradiated OP9 stromal cells (black) on signals from US7 ALL cells (red). Intact OP9 cells are very large and off-scale under the FSC/SSC voltage parameters used in a typical plot to gate for small compact ALL cells. Only cellular debris originating from OP9 cells are visible. Therefore OP9 signals do not contribute to the pErk or pMek values recorded for ALL cells.
(TIF)

S2 Fig Generation of pErk1/2 in serum-stimulated normal lymphocytes in LAX40 sample (A) and size differences in malignant lymphoblast population in LAX39 sample (B, C). Cells shown in panels A and B, C in S2 Fig were gated from the same analysis shown in panels B and C in Fig 5, respectively.
(TIF)

S3 Fig Phospho-flow analysis for Erk pathway inhibition by selumetinib in patient-derived and primary BCP ALL samples. (A, B) pre-B ALLs indicated in the panels were cultured for 24 hrs in αMEM + 20% FBS on OP9 stroma or in αMEM + 1% BSA (SFM, serum-free medium) without OP9 stroma, and treated for 4 hours with DMSO or 10 μM selumetinib. Cells were compared for pErk1/2 (A) or pMek (B) levels using BD antibodies. Results shown are representative of 2 independent experiments for TXL2, ICN06 and US7. Error bars, mean ± SD of 2 measurements performed on independent samples. *p<0.05; **p<0.01. (C, D) LAX57 and LAX56 (diagnosis and relapse samples, respectively) were cultured for 24 hours in medium with 20% FBS and OP9 stroma, or in medium with 1% BSA without stroma (SFM, serum-free medium), then analyzed for pErk1/2 (C) or pMek (D) using BD antibodies.
(TIF)
