# Supplementary figures and images for: Phosphoflow-Based Evaluation of Mek Inhibitors as Small-Molecule Therapeutics for B-Cell Precursor Acute Lymphoblastic Leukemia
Source: PLoS One. 2015 Sep 11;10(9):e0137917. doi: 10.1371/journal.pone.0137917 (PMC4567297; doi:10.1371/journal.pone.0137917)

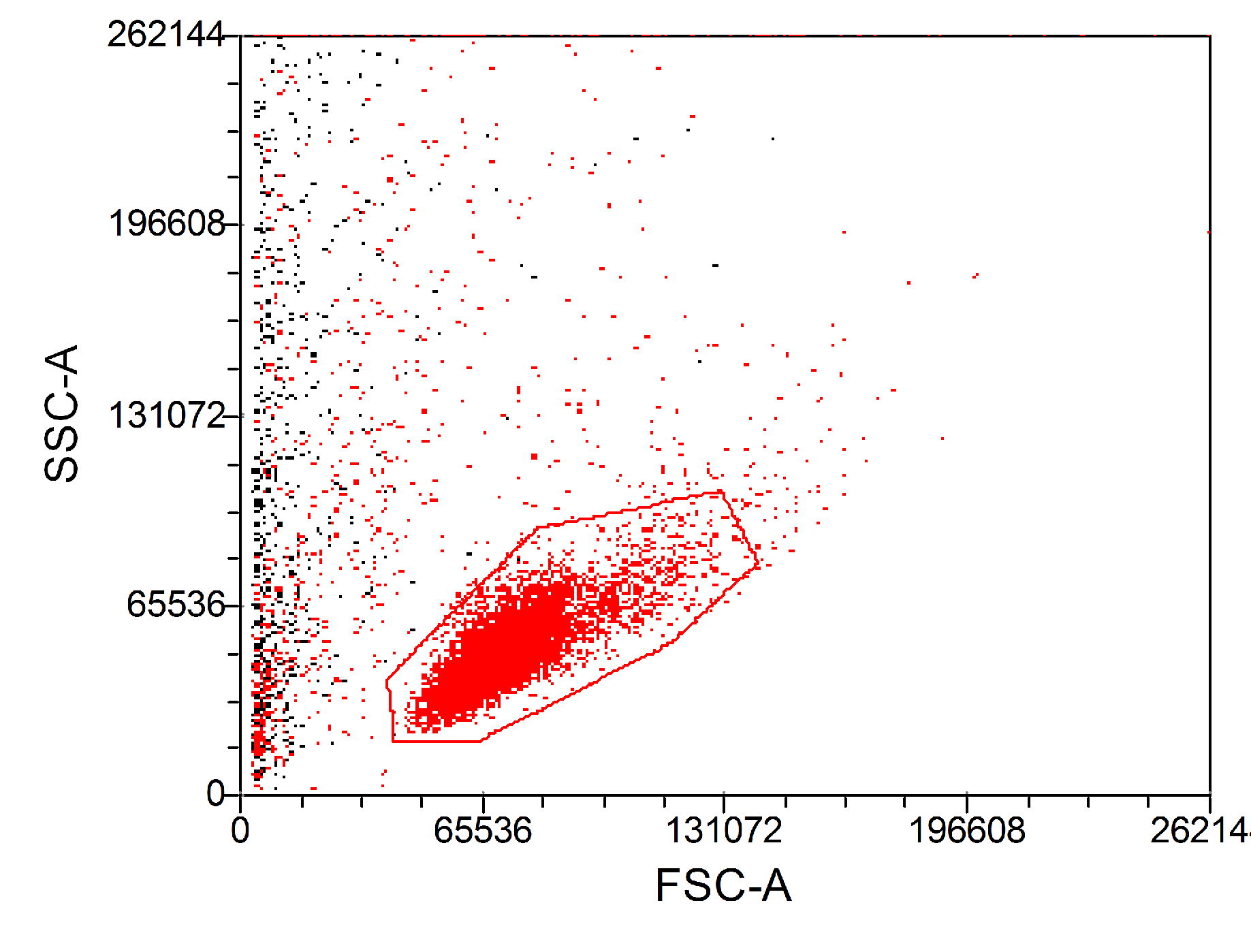

Supplement: S1 Fig — Intact OP9 cells are very large and off-scale under the FSC/SSC voltage parameters used in a typical plot to gate for small compact ALL cells. Only cellular debris originating from OP9 cells are visible. Therefore OP9 signals do not contribute to the pErk or pMek values recorded for ALL cells. (TIF) [file pone.0137917.s001.tif]

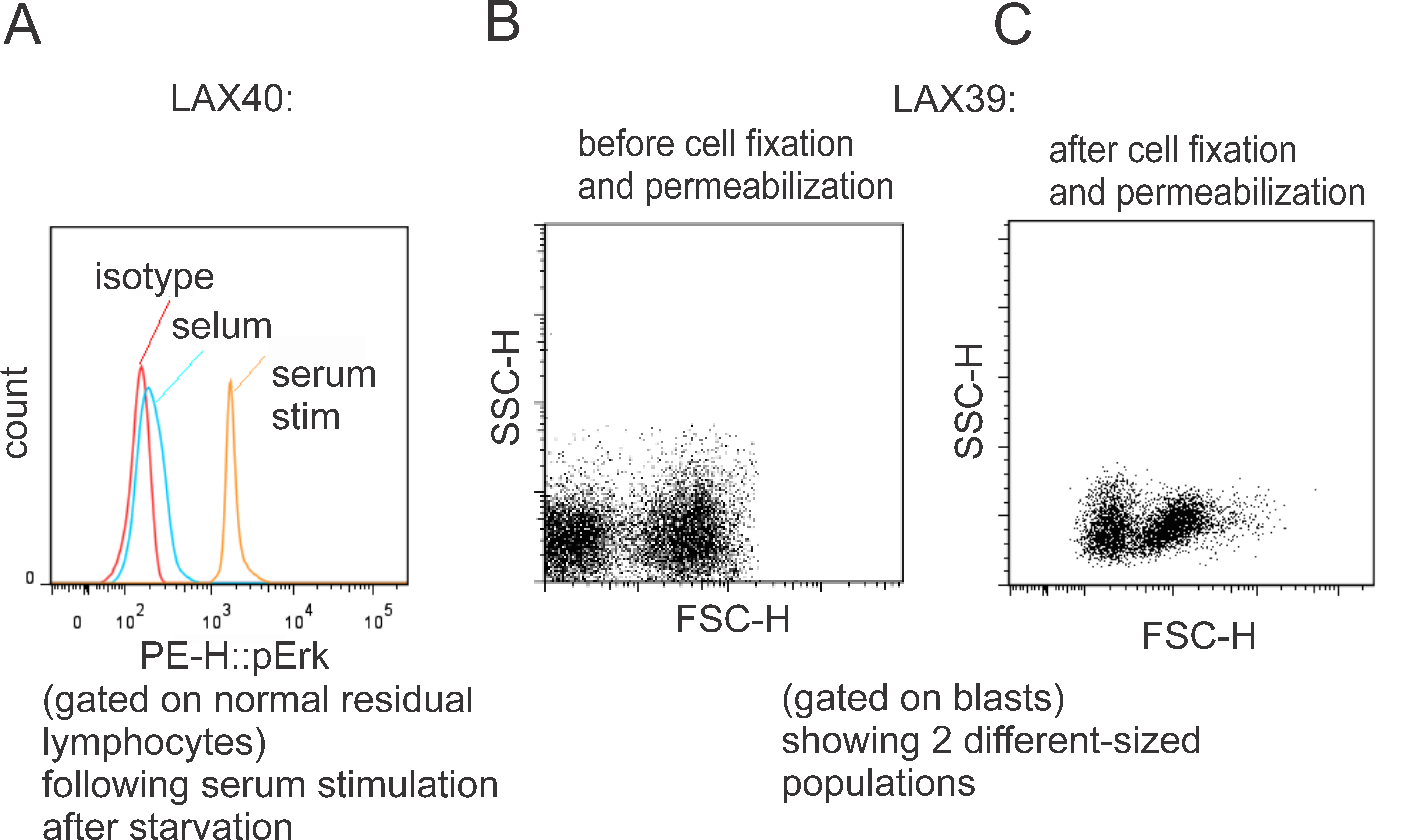

Supplement: S2 Fig — Cells shown in panels A and B, C in S2 Fig were gated from the same analysis shown in panels B and C in Fig 5, respectively. (TIF) [file pone.0137917.s002.tif]

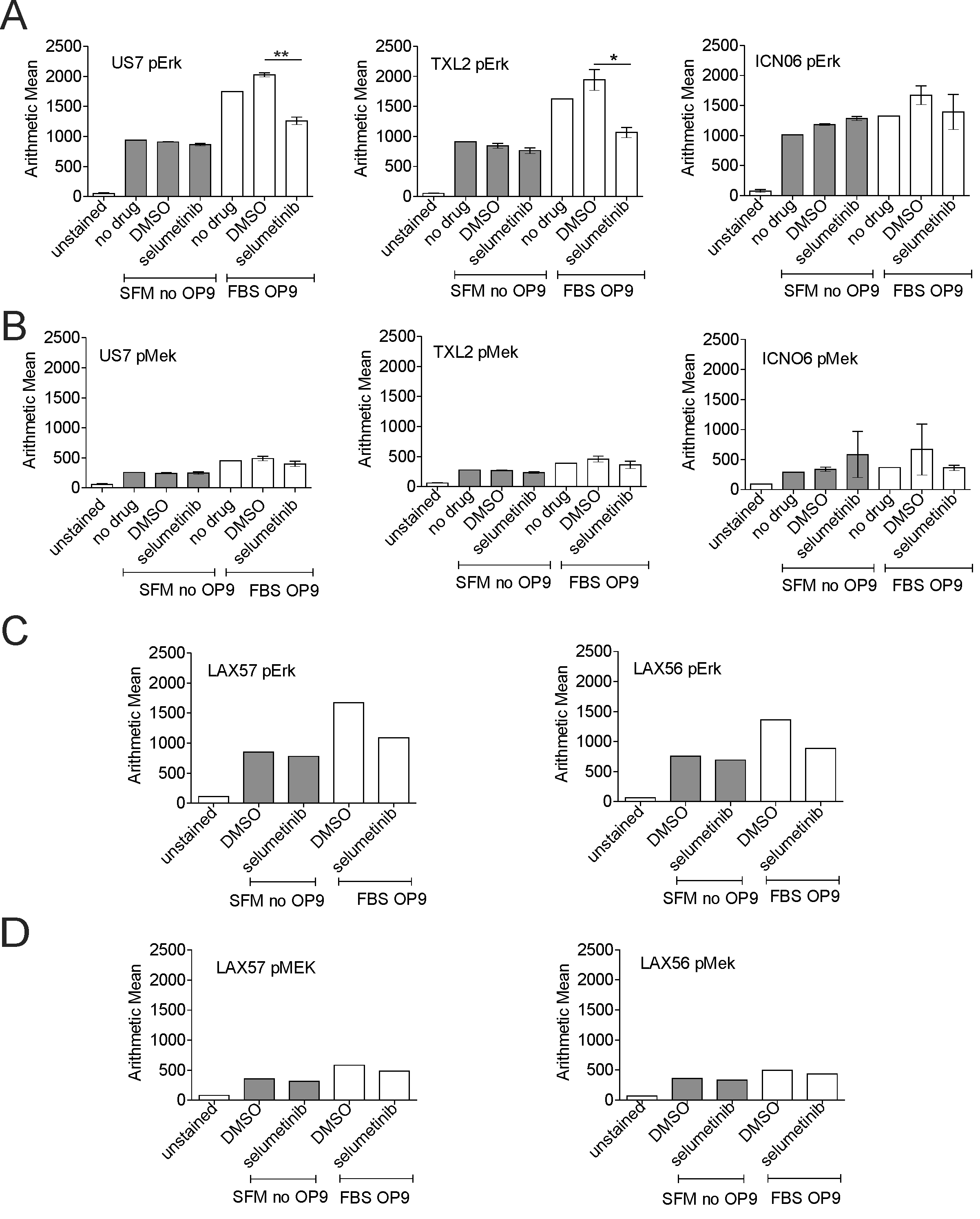

Supplement: S3 Fig — (A, B) pre-B ALLs indicated in the panels were cultured for 24 hrs in αMEM + 20% FBS on OP9 stroma or in αMEM + 1% BSA (SFM, serum-free medium) without OP9 stroma, and treated for 4 hours with DMSO or 10 μM selumetinib. Cells were compared for pErk1/2 (A) or pMek (B) levels using BD antibodies. Results shown are representative of 2 independent experiments for TXL2, ICN06 and US7. Error bars, mean ± SD of 2 measurements performed on independent samples. *p<0.05; **p<0.01. (C, D) LAX57 and LAX56 (diagnosis and relapse samples, respectively) were cultured for 24 hours in medium with 20% FBS and OP9 stroma, or in medium with 1% BSA without stroma (SFM, serum-free medium), then analyzed for pErk1/2 (C) or pMek (D) using BD antibodies. (TIF) [file pone.0137917.s003.tif]
